# SMRT Sequencing of Long Tandem Nucleotide Repeats in SCA10 Reveals Unique Insight of Repeat Expansion Structure

**DOI:** 10.1371/journal.pone.0135906

**Published:** 2015-08-21

**Authors:** Karen N. McFarland, Jilin Liu, Ivette Landrian, Ronald Godiska, Savita Shanker, Fahong Yu, William G. Farmerie, Tetsuo Ashizawa

**Affiliations:** 1 Department of Neurology and The McKnight Brain Institute, University of Florida, Gainesville, Florida, 32610, United States of America; 2 Lucigen Corporation, Middleton, Wisconsin, 53562, United States of America; 3 Interdisciplinary Center for Biotechnology Research, University of Florida, Gainesville, Florida, 32610, United States of America; The University of Queensland, AUSTRALIA

## Abstract

A large, non-coding ATTCT repeat expansion causes the neurodegenerative disorder, spinocerebellar ataxia type 10 (SCA10). In a subset of SCA10 patients, interruption motifs are present at the 5’ end of the expansion and strongly correlate with epileptic seizures. Thus, interruption motifs are a predictor of the epileptic phenotype and are hypothesized to act as a phenotypic modifier in SCA10. Yet, the exact internal sequence structure of SCA10 expansions remains unknown due to limitations in current technologies for sequencing across long extended tracts of tandem nucleotide repeats. We used the third generation sequencing technology, Single Molecule Real Time (SMRT) sequencing, to obtain full-length contiguous expansion sequences, ranging from 2.5 to 4.4 kb in length, from three SCA10 patients with different clinical presentations. We obtained sequence spanning the entire length of the expansion and identified the structure of known and novel interruption motifs within the SCA10 expansion. The exact interruption patterns in expanded SCA10 alleles will allow us to further investigate the potential contributions of these interrupting sequences to the pathogenic modification leading to the epilepsy phenotype in SCA10. Our results also demonstrate that SMRT sequencing is useful for deciphering long tandem repeats that pose as “gaps” in the human genome sequence.

## Introduction

Spinocerebellar ataxia type 10 (SCA10) is caused by the expansion of a pentanucleotide repeat region within intron 9 of *ATAXIN 10* [1]. This mutation belongs to a class of non-coding repeat expansions that cause other spinocerebellar ataxia disorders (SCA8, SCA12, SCA31, SCA36) as well as amyotrophic lateral sclerosis/frontotemporal dementia (ALS/FTD), myotonic dystrophy types 1 (DM1) and 2 (DM2), fragile X-associated tremor/ataxia syndrome (FXTAS), Friedreich’s ataxia (FA), and Huntington’s Disease Like 2 (HDL2). For the majority of these disorders, the large expansion size prohibits complete sequencing across the entire expansion.

In SCA10, the pathogenic alleles range from 280 to 4,500 repeats with a mean expansion size of 2071 repeats [2]. While the expansion was originally assumed to consist of a pure expansion sequence, subsequent studies have identified interruption motifs at the 5’ and 3’ ends of the SCA10 expansion in a small subset of SCA10 patients [3, 4] We postulate that these interruption motifs act as phenotypic modifiers, as they correlate with an increased chance of having epileptic seizures in addition to typical ataxic symptoms. Yet, our knowledge of the internal sequence of the SCA10 expansion is extremely limited by the technical and computational challenges. The expansion size is beyond the limits of analysis by conventional Sanger sequencing, which is generally limited to read lengths of ~1 kb. Conversely, next-generation sequencing (NGS) of the repeat expansions is a challenge, as assembly of NGS-derived short reads of a highly repetitive sequence is problematic. Advanced NGS technologies, such as TruSeq synthetic long-read sequencing method [5, 6], are also likely to face the same challenges as other short-read technologies.

Thus, we turned to the third-generation sequencing technology, Single Molecule Real-Time (SMRT) sequencing [7, 8], to sequence across the entire span of the SCA10 repeat expansion. We were attracted to SMRT sequencing by its extraordinarily long read lengths in the multi-kilobase range, which would be suitable for SCA10 expansions, as well as its past success with other repeat expansion disorders [9, 10]. Here we report successful sequencing across the expansion (ranging from 2.5 to 4.4 kb) in three SCA10 patients. Our data reveal novel interruption motifs within the interior of the repeat expansion, which would be useful in investigating the pathogenic role of such motifs in SCA10.

## Results

### SCA10 expansion templates for SMRT sequencing

**Fig 1:**
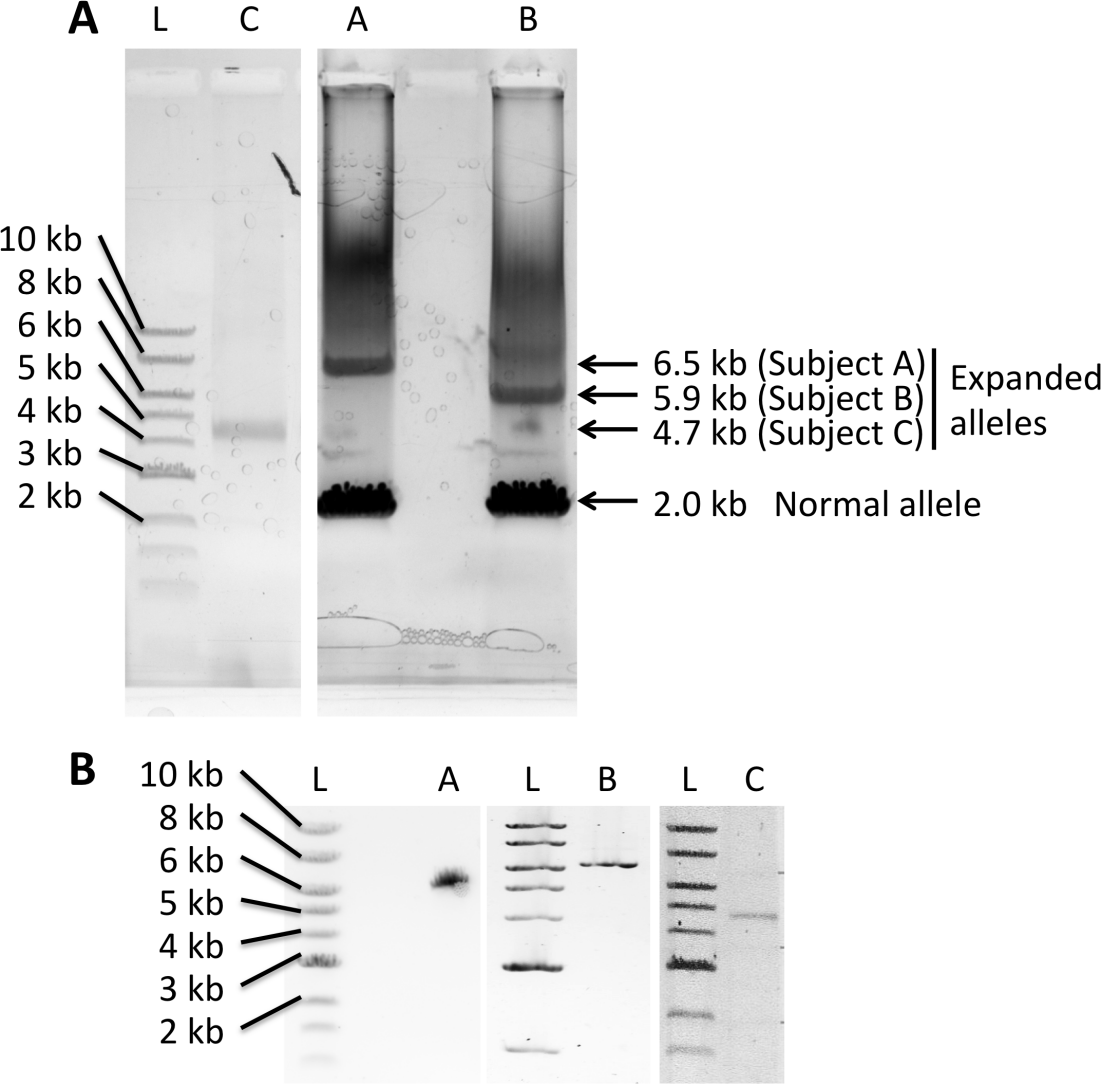
SCA10 expansion templates for SMRT sequencing. (A) PCR amplification of the SCA10 expansion from gDNA extracted from blood lymphocytes (Subjects A and B) or from somatic cell hybrid lines (subject C). Lanes are cropped from non-adjacent lanes of the same gel. The full gel is shown is S1 Fig. Arrows indicate the size of bands that were excised for cloning and sequencing (subject A, the 6.5 kb band; subject B, the 5.9 kb band; subject C, the 4.7 kb band) (B) Purified template from cloned PCR products in Fig 1A for SMRT sequencing. L: 1 kb ladder (New England Biolabs). The full gels are shown in S2 Fig.

The three SCA10-positive individuals chosen for sequencing had differing clinical presentations and relatively short SCA10 expansions (1200, 844, and 800 penta-nucleotide repeat units for subjects A, B and C, respectively). Consensus sequences can be found in S1 File. We PCR amplified the SCA10 expansion using a modified extra-long PCR protocol, yielding PCR products ranging from 4.7 to 6.5 kb in length (Fig 1A). To increase the amount of input DNA available for library construction for SMRT sequencing, we cloned the SCA10 PCR products into a linear vector, pJAZZ-OCmin, for growth in *E*. *coli*. The resulting cloned DNA fragments were excised from the plasmid backbone, gel purified, and submitted for library construction and SMRT sequencing (Fig 1B). As there is no consensus or reference sequence for an “SCA10 expansion,” we do not have a reference to which to compare the sequences generated in this study. Therefore, as a surrogate for determining the overall accuracy in sequencing results, we examined the adjacent, flanking sequences on the 5’ and 3’ ends of the expansion and examined their accuracy by comparison to the reference genome sequence in that region (Table 1). All sequence alignments from both 5’ and 3’ flanking regions, as well as the extreme 5’ and 3’ regions of the repeat expansion, in all three subjects resulted in 98–100% identity. Sequence results from within the expansion itself are likely to be highly comparable thus establishing a high degree of reliability in the resulting sequence.

**Table 1:** SMRT sequencing results summary.

	Subject A		Subject B		Subject C	
	Gel Estimated^§^	SMRT sequence	Gel Estimated^§^	SMRT sequence	Gel Estimated^§^	SMRT sequence
**Template Size**	6500 bp (~840 rpts)	6475 nts (870 rpts^**^**^)	5900 bp (~820 rpts)	5951 nts (884 rpts^**^**^)	4700 bp (~530 rpts)	4476 nts (514 rpts^**^**^)
**5’ Flanking region** *		99.9% identity of 1038 bp		99.6% identity of 904 bp		99.1% identity of 650 bp
**5’ end of SCA10 expansion** ^**¶**^		98.5% identity of 796 bp		98.4% identity of 733 bp		98.2% identity of 935 bp
**3’ end of SCA10 expansion** ^**¶**^		100% identity of 626 bp		99.5% identity of 485 bp		99.7% identity of 350 bp
**3’ flanking region** *		99.6% identity of 564 bp		99.1% identity of 1297 bp		99.1% identity of 454 bp

^§^as estimated by gel electrophoresis of cloned expansion fragment excised from plasmid backbone.

^**¶**^ **As compared with Sanger sequencing.**

*Alignment based on reference genome sequence (NC_000022.11; GI: 568815576, Region: 45794523..45796589) using LALIGN.

**Fig 2:**
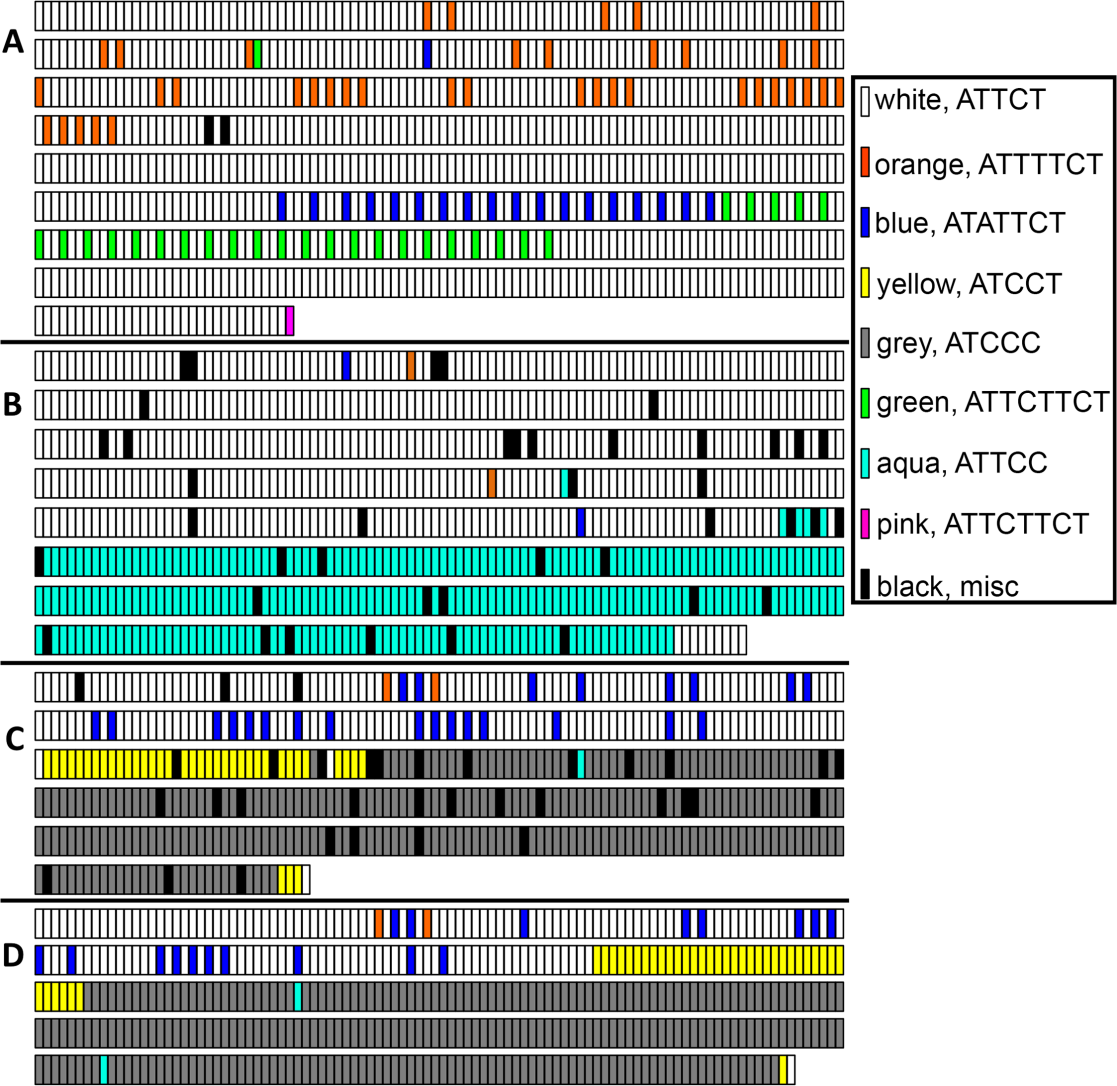
Schematic representations of the repeat expansions. (A) SCA10 expansion in subject A. (B) SCA10 expansion in subject B. (C) SCA10 expansion in subject C. Rectangles represent sequence motifs, as indicated by the color key, in the 5’ (upper left) to 3’ (lower right) direction. Black rectangles indicate unverified motifs described further in Fig 3B and are indicated as follows: A: ATTTCT, ATTTCT; B: ATTTCT, A, ATTTCT, A, ATTCCT, TAC, ATTTCT, A, ATT, ACTTCT, ATTCA, ATTTCT, ATTTCT, T, ACTTTCT, TCTTTCT, ATTT, ATTTCT, ATCT, ATTTCT, ATTTCT, ATTTCT, ATTTCT, ATTTCT, T, ATCC, ATTC, ATTTCC, C, ATTTCC, TTCCC, ATTTCC, CATCC, ATTTCC, C, C, C, C, ATTC, ATTTCC, ATTCC; C: ATCT, ATCT, ATCT, AT, ATCT, T, ATC, ATCC, ATCC, ATCC, ATCC, ATCC, ATCC, ATCC, ATCC, ATCC, C, ATCC, ATCC, ATCC, ATCC, ATCC, ATCC, ATCC, ATC, ATCC, ATCC, ATCC, ATCC, ATCC, ATCC, ATCC, ATCC, C

^as determined by counting motif blocks in Fig 2. Rpts, repeats; nts, nucleotides; bp, base pairs

### SCA10 expansion—repeat motif composition

**Fig 3:**
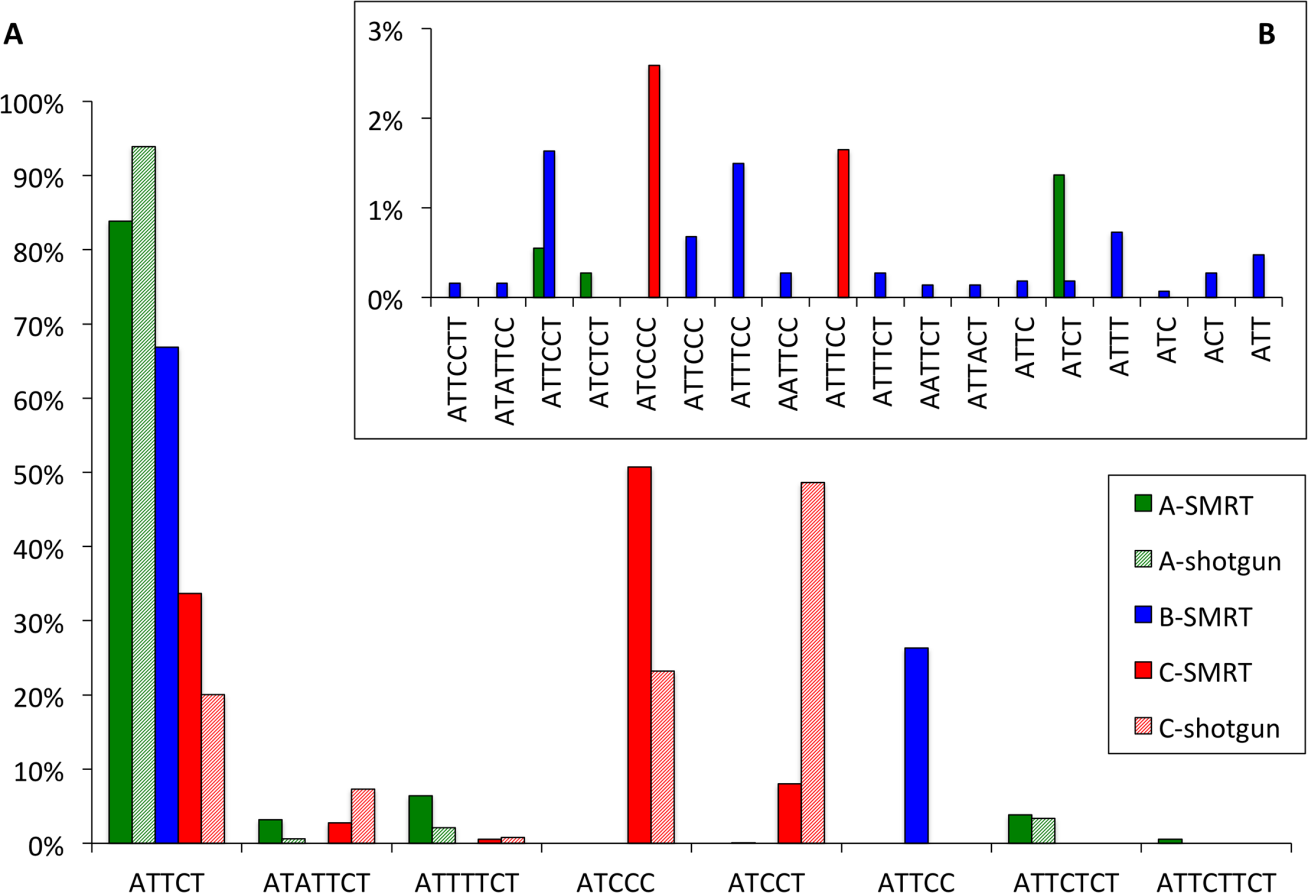
Proportion of repeat motifs in SCA10 expansions. Proportions are calculated as the percentage of nucleotides of each motifs divided by the total number of nucleotides for each expansion. Motifs present in SMRT sequence results that are verified by Sanger sequencing methods (“shotgun”) comprise the majority of motifs seen (A) while some motifs are unverified (B). Green, SMRT sequencing results from subject A; Green hatched, random shotgun sequencing results from subject A; Blue, SMRT sequencing results from subject B; Red, SMRT sequencing result from subject C; Red hatched, random shotgun sequencing results from subject C.

As expected, we found the canonical ATTCT motif in all three SCA10 expansions (Fig 2A–2C). However, the composition of ATTCT motifs within each expansion varied dramatically. Of the three samples sequences, subject A had the largest number of ATTCT motifs (white rectangles in Fig 2), representing 84% of the total repeats; in comparison, subjects B and C had 67% and 34% ATTCT repeats, respectively (Fig 3). Beyond the typical ATTCT motif, all three SCA10 subjects had varying degrees of interrupting motifs. Some of these interruption motifs are found uniquely in only one subject (e.g., ATTCTTCT and ATTCTCT in subject A, ATTCC in subject B, and ATCCC and ATCCT in subject C). Other interruption motifs were shared in two of the three expansions (e.g. ATATTCT and ATTTTTCT in subject A and C). We are able to verify the presence of these seven interruption motifs by Sanger sequence obtained from the extreme ends of the each expansion, as well as by examining random shot-gun cloned products of the expansion [11]. Thus, we are confident that these interruption motifs are not the result of sequencing or assembly errors.

In addition to these major interruption motifs, several other rare interruption motifs were found (Fig 3B). All together, these rare interruption motifs accounted for 2.2%, 6.8%, and 4.2% of the total expansion sequence in subjects A, B and C. The proportion of any individual minor interruption motif was below 3% of the total number of repeat units. Furthermore, some of these were seen only once or twice throughout the expansion. The validity of these minor motifs has not been verified either by Sanger sequencing or by our random shot-gun cloning. The error propensity of SMRT sequencing skews towards single base pair insertions [12]. Thus, the minor hepta- and hexanucleotide interruption motifs may simply be artifacts of SMRT sequencing. Conversely, the minor interruption tri- and tetranucleotide motifs (ACT, ATC, ATT, ATCT) may represent deletion errors in SMRT sequencing, the second most common form of errors in SMRT sequencing [12].

### SCA10 expansion—repeat motif structure

The expansion of subject A contains the most ATTCT motifs of the three expansions sequenced (Fig 2, white rectangles). We also observed repetitive blocks of [ATTTTCT ATTCT] (orange, white) as well as of [ATATTCT ATTCT ATTCT] (blue, white, white). These sequence patterns were confirmed by sequencing random shot-gun clones of the expansion from this individual [11]. The ATTTTCT and ATATTCT heptanucleotide motifs have been described previously at the 5’ end of other expansions (e.g., subject C) [2, 3]; however, the difference in individual A is the increased frequency of these motifs [11] (Fig 2A). In addition, we observed two unique motifs—the heptanucleotide motif ATTCTCT (green) and the octanucleotide interruption motif ATTCTTCT (pink). Both of these motifs were observed in our prior approach of random shot-gun cloning of the expansion [11]. The ATTCTCT motif is seen in repetitive blocks interspersed [ATTCTCT ATTCT ATTCT] (green, white, white). The ATTCTTCT motif was only once at the 3’ end of the expansion.

Sequencing the SCA10 expansion in Subject B revealed an ATTCC motif that encompassed nearly one-third of the entire expansion (Fig 2B, aqua). The 5’ end of this expansion is composed primarily of typical ATTCT repeats, whereas the 3’ end is composed of ATTCC motifs. Random fragment sequencing of this expansion following previous methods [11] in addition to Sanger sequencing of the 3’ end of this expansion confirmed the presence of these interruptions (data not shown). Of note, this motif was previously identified in SCA31 expansions (identified as TGGAA) [13].

The expansion of subject C is well studied of amongst all SCA10 expansions. Interruption motifs were first identified in this family and then detected by the “ATCCT-PCR” in other SCA10 families [2, 3]. The expansion in this individual has the characteristic block of [ATTTTCT ATTCT ATATTCT ATTCT ATATTCT ATTCT ATTTTCT] (orange, white, blue, white, blue, white, orange) at the 5’ end of the expansion (Fig 2C). We also observed numerous ATCCT motifs (yellow) near the midpoint of the expansion and three copies near the 3’ end. Notably, the 3’ end of this expansion is composed primarily of ATCCC motifs (grey). These interruption motif is associated with a high risk of epilepsy in carrier SCA10 patients. Furthermore, expansions that carry interruption motifs exhibit paradoxical anticipation during intergenerational transmission of the disease allele—that is, intergenerational length contractions of the interrupted SCA10 allele are associated with progressively earlier onset of the disease in successive generations (contrary to what is seen for other repeat expansion disorders) [2, 4].

A second subclone of subject C was sequenced using an advanced version of sequencing chemistry, C4. While the sequencing error rate is similar to the C2 chemistry used on the other sequences, the improved chemistry allows for longer average sequencing reads. When applied to our second subclone, we found that there were greater than 106 sequencing reads that were each over 20 kb in length. Realizing that these reads were likely multi-pass sequencing reads that passaged across the SMRT bell adaptors many times, we examined the 26 reads with 7 or more sequencing passes having a minimum mean predicted accuracy of >99%. Each multi-pass sequencing read is derived from a single ZMV (single individual DNA molecules) and represents the consensus of that single DNA molecule. With this data, we took two approaches. We first examined the alignment of the 26 reads to evaluate the effect of the DNA preparation method on the resulting DNA sequence reads. The SCA10 expansion was prepared from a single isolated bacterial colony and then grown in a liquid culture for the plasmid preparation. Thus, as variability seen between the individual DNA sequences likely results from changes that occur during growth in bacteria. We see that the greatest variability and stutter between sequencing reads is in the 3’ region which contains the ATCCC repeats (see the alignment in the S2 File). In the second approach, we used the 26 multi-pass reads to generate a consensus (Fig 2D). The overall structure of the two subclones (Fig 2C versus 2D) is very similar. There are improvements in the 3’ end sequence (fewer questionable, “miscellaneous” motifs, black rectangles); however, the interruption structure in the 5’ end varies between the two subclones and likely represents mutation events during bacterial growth.

## Discussion

SCA10 is one of over 20 neurological diseases caused by repeat expansion mutations and one of 10 where the expansion is a non-coding repeat. Though not fully understood, disease pathogenesis is thought to involve an RNA-based gain-of-function mechanism, in which RNA transcripts of the repeat expansion accumulate and sequester critical RNA binding proteins (e.g., hnRNP K [14–16]) resulting in their loss of function. The epilepsy phenotype in a subset of SCA10 patients highly correlates with the presence of the ATCCT-type repeat interruptions [4], which is also found at the 5’ end of subject C in this paper. Furthermore, the typical inverse correlation between repeat length and age at onset seen in ATCCT-negative SCA10 expansions is absent in those individuals that carry these ATCCT repeat interruptions (which also include the ATTTTCT, ATATTCT, and ATCCC motifs) [2]. Thus, as RNA binding proteins interact with their target RNAs in a sequence-specific fashion, the composition of the expansion sequence would be a critical component in the pathogenic RNA-gain-of-function mechanism in SCA10.

To understand the structure and overall repeat composition of the SCA10 expansion, we turned to the single-molecule real-time (SMRT) sequencing technology to gather sequence information across the entire span of these interruptions. The benefit of using SMRT sequencing lies in its ability to obtain long contiguous sequences across the entire length of the expansion. We utilized this feature of SMRT sequencing to generate consensus sequences from multiple reads of each expansion. While there are certainly errors in the sequence that likely result from either PCR stutter during amplification (which can be seen even when amplifying the normal allele [1]), E. coli induced changes during cloning, sequencing and/or assembly, we have a high degree of confidence in the resulting consensus expansion sequences. While we cannot call these sequence 100% accurate, in the absence of a reference SCA10 expansion sequence, these data give us a proof of concept and good foundation upon which we can design further experiments to improve upon these results.

We believe that the strategy outlined for the SCA10 expansion will be generally applicable to other repeat expansion disorders. However, as this approach relies on generating sufficient quantities of DNA for subsequent sequencing, the weakness of our approach lies on the necessity of PCR-amplifying and cloning of the repeat expansion. Recent improvements in SMRT sequencing chemistry and instrumentation may abrogate these limitations as newer versions of SMRT sequencing chemistry have reduced the amount of DNA needed for input (down to 0.5 micrograms in the C4 version versus 5 micrograms in the C2 version). Additionally, the MinION device by Oxford Nanopore Technologies, a single-molecule sensing device, holds promise for long-read sequencing microsatellite expansions. Within the device, a DNA processive enzyme pulls a single DNA strand through a biological nanopore with an accompanying electrical sensor. As each nucleotide is pulled through, characteristic changes in the electrical current are used to determine the DNA sequence. Enhancements in the MinION chemistry and base-calling algorithms are promising but not yet widely available [17, 18], yet we anticipate further progress with this technology.

From the limited sampling of these SCA10 expansions, our SMRT sequencing reveals strikingly different structures of interruption motifs between study subjects. While these three subjects have differing clinical presentations, the relationship between expansion structure and clinical phenotype remains to be seen. However, our results highlight the importance of the sequence composition of repeat expansions and the utility of SMRT sequencing for delineating the differences between expansions.

## Methods

### SCA10 subjects and DNA preparation

Samples were obtained following written, informed consent under protocols approved by local Institutional Review Boards (IRBs) at the University of Texas Medical Branch and the University of Florida which specifically approved this study. We selected three SCA10 subjects. Briefly, subject A is a North American Sioux Indian descendent with SCA10 who developed mild ataxia in his late 80s without accompanying epilepsy [19]. Subject B is an asymptomatic carrier of the SCA10 repeat expansion in her late 50s from a Mexican SCA10 family whose affected members exhibited cerebellar ataxia without epilepsy. Subject C is a Mexican-American SCA10 patient with both ataxia and epileptic seizures that began in his early 30s. The SCA10 expansions in subjects A, B, and C contained 1400, 840, and 800 repeats, respectively, in blood lymphocytes. The SCA10 expansions in subjects A and C have been partially analyzed by Sanger sequencing of random DNA fragments of PCR products amplified from blood leucocytes [11]. In subject C, the PCR product was amplified from the genomic DNA of somatic cell hybrid line [20] derived from the subject’s lymphocytes.

### Long-range PCR

Long-range PCR was carried out using the GeneAmp XL PCR kit (Life Technologies) in a 50 μL reaction volume containing 200 μM of dNTPs, 1.25 mM of MgSO_4_ 400 nmol each of primers, 200 ng of high molecular weight genomic DNA as template, and 1 unit of rTth DNA polymerase. PCR primers (F, 5’-ATAACCAGCTTTTGGTTTTGGCTAATCT-3’ and R, 5’-AGGAGAGAAATTTTACAGCTGGGTCTCT-3’) were designed to anneal to non-repeat regions flanking the repeat with 832 and 1165 bp of upstream and downstream sequence, respectively, based on the human genomic sequence data (Accession Number Z84478). PCR conditions consisted of an initial denaturation step at 94°C for 1 min followed by 35 cycles of a three-step amplification cycle of: 94°C denaturation for 15 sec, 61°C annealing for 30 sec, and 64°C extension for 15 min with the addition of 20 second increments to the extension time of each cycle between cycles 16 to 35. PCR products were electrophoresed on 0.5% agarose gels and visualized by SYBR Safe (Life Technologies). PCR products were purified from the gel using the Wizard SV Gel and PCR clean-up kit (Promega, Madison, WI) according to manufacturer’s instructions.

### Direct sequencing of the 5’ and 3’ ends of the expansion alleles

Using sequencing primers complementary to the 5' or 3' unique sequences flanking the repeat, the purified PCR products were sequenced by the Sanger method at the Interdisciplinary Center for Biotechnology Research (ICBR), Sanger Sequencing core facility at the University of Florida. Typical reads often extended up to 200 pentanucleotide repeats (or about 1kb) from each end. DNA sequences obtained from this method were used to partially confirm the consensus sequences spanning the SCA10 expansion obtained by SMRT sequencing.

### Linear vector cloning

**Fig 4:**
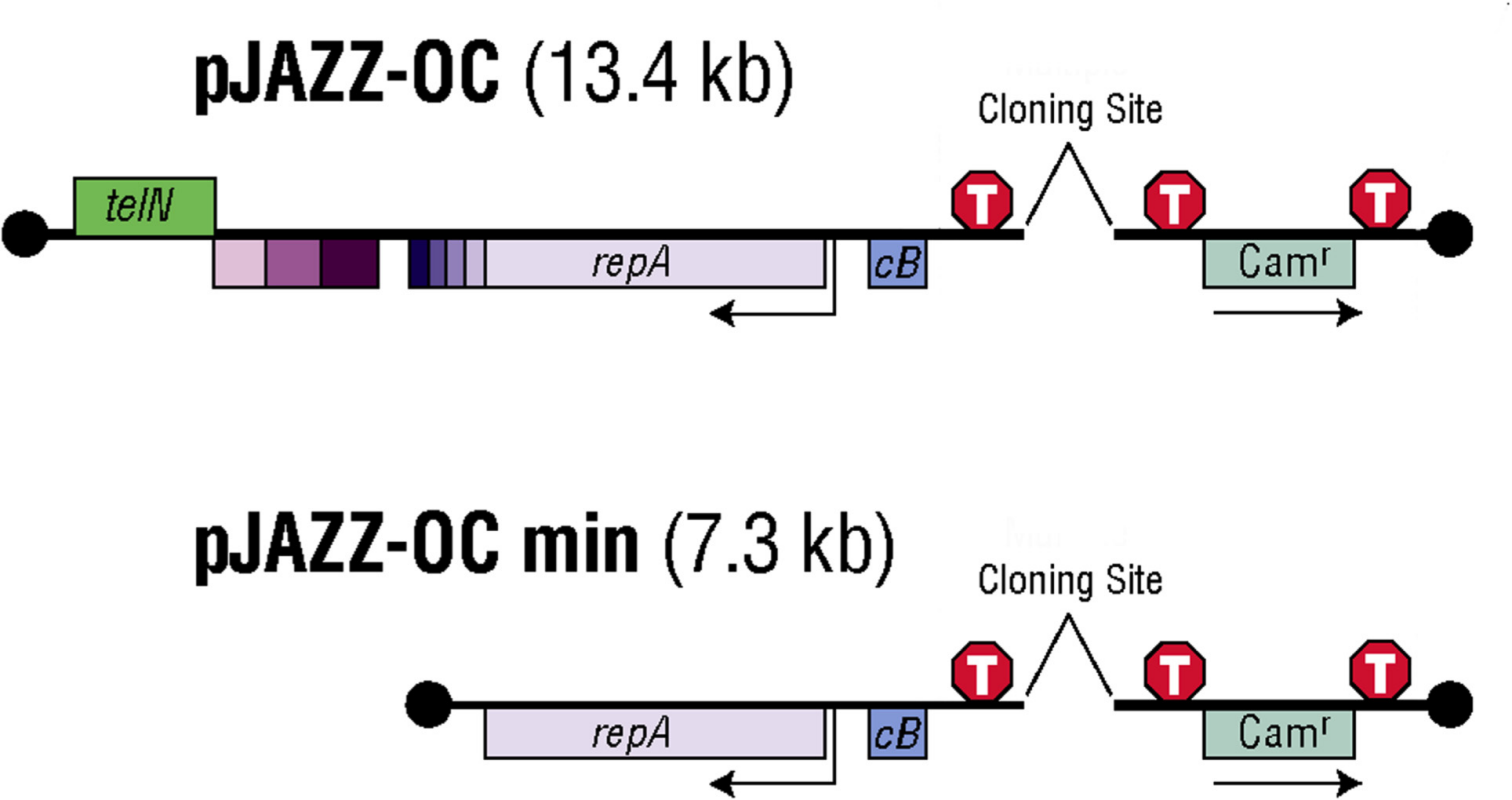
pJAZZ-OCmin vector. The telN gene and several non-essential genes from phage N15 (purple boxes) were deleted from the vector pJAZZ-OC. repA, cB: replication protein genes; Cam^r^, chloramphenicol resistance; T, terminator. Closed terminal hairpin structures are indicated by black circles.

Purified DNA products were cloned into pJAZZ-OCmin, a derivative of the linear pJAZZ-OC vector (Lucigen, Middleton, WI), which is optimal for cloning unstable DNA fragments (Fig 4; [21]). pJAZZ-OCmin lacks several genes of the parental vector, most notably telN, which is responsible for maintenance of the linear structure [22]. TelN activity is provided in *trans* by the *E*. *coli* host strain TSA G5, which is a TelN-overexpressing derivative of the BigEasy TSA strain (Lucigen). The remaining N15 genes that were deleted in the construction of pJAZZ-OCmin are not essential for plasmid function [21, 22]. To prepare the PCR product for cloning, it was blunt ended using the Quick Blunting Kit (New England Biolabs, Ipswich, MA) and ligated to the left and right arms of the plasmid backbone, with subsequent transformation into TSA G5 electrocompetent cells. Transformed clones were selected with 12.5 microgram/ml chloramphenicol and grown at 16°C to maintain stability of the expanded repeat. Several independent transformants were chosen for further analysis of insert size via gel electrophoresis of the cloned insert. The clone containing the longest repeat expansion was selected for SMRT sequencing.

### SMRT sequencing and data analysis

The DNA fragment containing the repeat was released from the vector using restriction endonuclease digestion and was gel purified as described above. Five micrograms of linear DNA fragment containing the SCA10 repeat expansion was submitted to the NextGen DNA Sequencing core at the University of Florida’s Interdisciplinary Center for Biotechnology Research. SMRTbell adaptors were ligated to the linear template DNA and purified using the RS DNA Template Preparation Kit 2.0 (Pacific Biosciences, Menlo Park, CA) according to the manufacturer’s instructions. Purified template was loaded onto a single SMRT sequencing cell (Pacific Biosciences), and sequencing runs were performed on the PacBio RS instrument with a 75 min sequencing time using C2 chemistry. A second subclone of subject C was sequenced with a 90 min sequencing time using C4 chemistry.

### Sequence data analysis and consensus assembly of SCA10 expansion sequence

An initial filter was applied to SMRT sequencing reads to remove short sequencing reads less than 50 nts in length and with a quality score of less of 0.75. Then, SMRT sequencing reads with read-lengths longer than 4 kb and a quality score greater than 0.85 were assembled using Allora, an overlap-layout-consensus algorithm suitable for *de novo* assembly of long sequence reads developed by Pacific Biosciences. The consensus sequence output from the Allora assembly was used as a reference for the initial round of assembly refinement using the Basic Local Alignment with Successive Refinement (BLASR) algorithm [23]. In the first round, sequence read lengths greater than or equal to 4 kb in length with quality scores greater than 0.85 were mapped to the Allora reference assembly. The output from the first round of BLASR mapping was used as a refined reference sequence in a second round of BLASR mapping. The second round used only read lengths longer than 4.5kb with quality scores greater than 0.80. Multiple rounds of mapping were used to refine the consensus sequence for each SCA10 expansion in order to reduce the number of nucleotide sequence discrepancies. The sequence from the second round of BLASR mapping was evaluated for motif composition. Final version of each sequence have been submitted to Genbank under accession numbers KM610327 (Subject A), KM610328 (Subject B) and KM610329 (Subject C).

### Sequence verification

We verified SMRT-generated SCA10 expansion consensus sequence in three ways. First, we aligned the SMRT consensus sequence with Sanger sequences at the beginning and end of repeats of each template. Second, we compared the composition of the repeat motifs with results that we have generated by random DNA fragmentation and subcloning of the SCA10 expansion [11]. Finally, we compared the flanking sequence surrounding the repeats to the reference genome sequence. For sequence comparisons, we used LALIGN [24] to generate pair-wise alignments between the SMRT and Sanger sequences (or reference genome sequences for flanking regions).

### PacBio raw sequence file upload

Raw sequence (hdf5) files are uploaded to the NCBI’s Sequence Read Archive (SRA) under the following BioProject ID PRJNA287678 (accession number SRP059742). submission IDs: Subject A, SRX1067754; Subject B, SRX1075640; Subject C-clone 1, SRX1076975; Subject C-clone 2- SRX1076989.

## Supporting Information

S1 Fig Original gel images of Fig 1A. A, B, C and L labels are as in Fig 1. Bars above the lane indicate areas of the gels not shown in Fig 1A.
(TIF)

S2 Fig Original gel images of Fig 1B. Labels are as in Fig 1.
(TIF)

S1 File Consensus sequences of each clone. Corresponding Genbank accession number are included with the corresponding sequence. Sequences include upstream and downstream sequences in intron nine of *ATXN10* surrounding the SCA10 expansion.
(DOCX)

S2 File Multipass sequence aligments. Alignment of the 26 sequences with multipass sequence reads of greater than 7 passes (from the sequencing of the second clone from subject C).
(TXT)
