# Supplementary figures and images for: SMRT Sequencing of Long Tandem Nucleotide Repeats in SCA10 Reveals Unique Insight of Repeat Expansion Structure
Source: PLoS One. 2015 Aug 21;10(8):e0135906. doi: 10.1371/journal.pone.0135906 (PMC4546671; doi:10.1371/journal.pone.0135906)

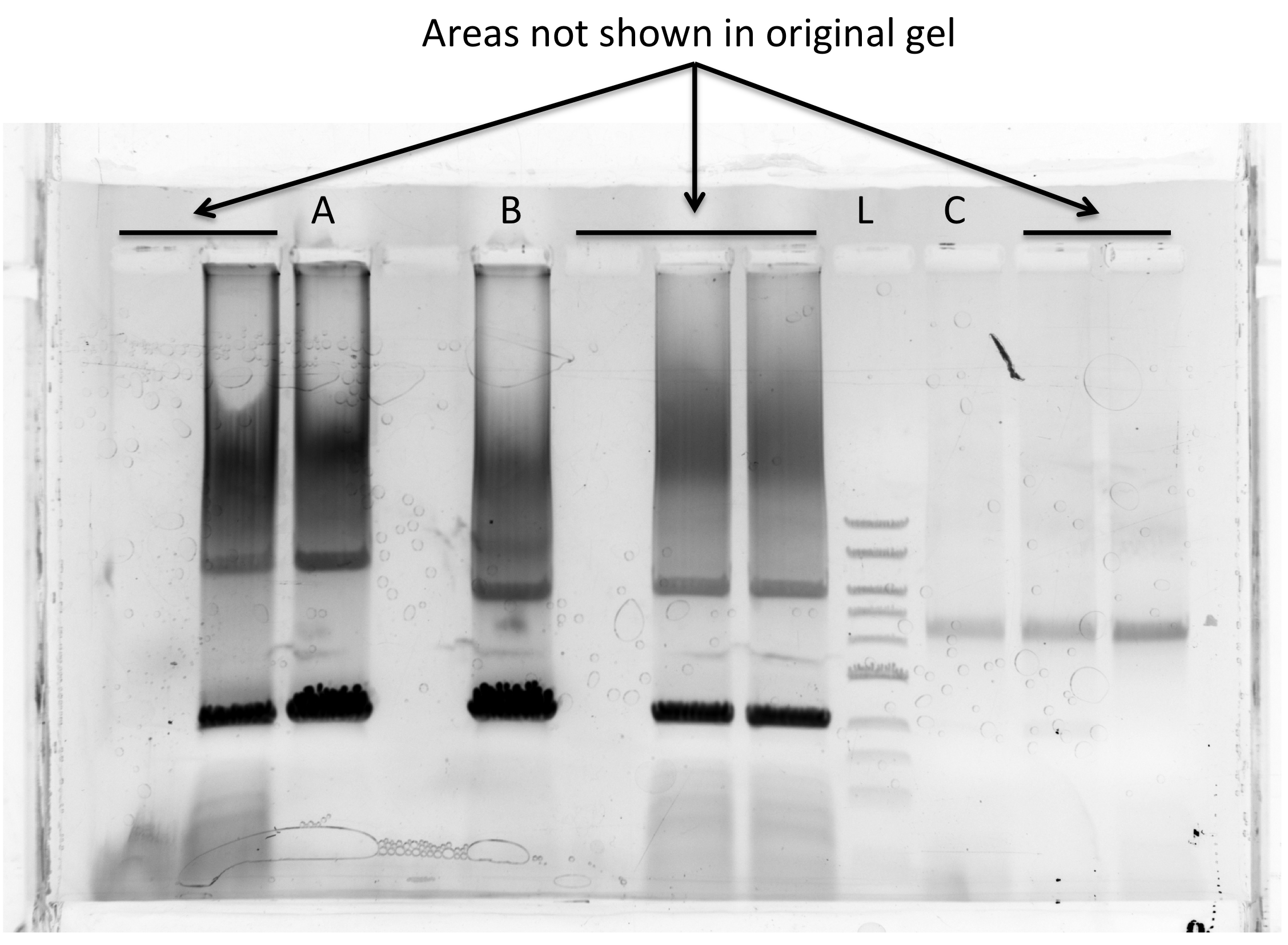

Supplement: S1 Fig — A, B, C and L labels are as in Fig 1. Bars above the lane indicate areas of the gels not shown in Fig 1A. (TIF) [file pone.0135906.s001.tif]

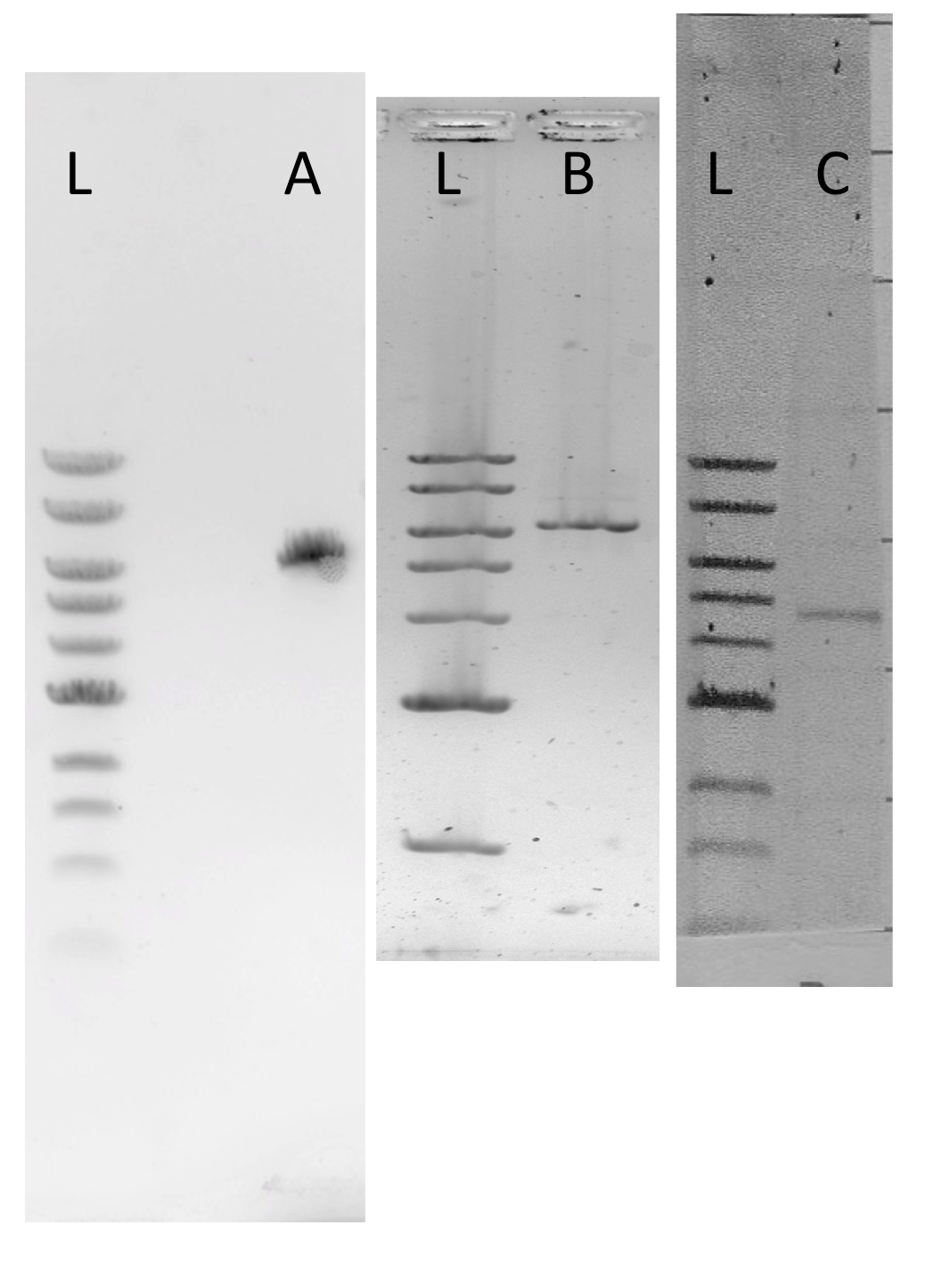

Supplement: S2 Fig — Labels are as in Fig 1. (TIF) [file pone.0135906.s002.tif]
