# Supplementary material for: SMRT Sequencing of Long Tandem Nucleotide Repeats in SCA10 Reveals Unique Insight of Repeat Expansion Structure
Source: PLoS One. 2015 Aug 21;10(8):e0135906. doi: 10.1371/journal.pone.0135906 (PMC4546671; doi:10.1371/journal.pone.0135906)
Supplement: S1 File — Corresponding Genbank accession number are included with the corresponding sequence. Sequences include upstream and downstream sequences in intron nine of ATXN10 surrounding the SCA10 expansion. (DOCX) [file pone.0135906.s003.docx]

>KM610327 (Subject A)

TAATTCACTGACTTTCTTGCCTGTCAGTTTATAAGAACAAAGATAAAAATGATAACAGGCTTTCCTTCAGAAATGATGCAAGACAGGAATCAATGGAATGACATCTTAAAAATACTAAAGGAAAACTGTTAATCTAGAATTATGTATCCAGCCAAAATATCCTTCAAAAATGAAGGTCAAAAAAAAGAAGGTAAAATGTCCTGTAAGAAACGTTAAATGAAGTTCAACAAGGTGAAAGTAAATGATAGCACATGGAAACCTGACCTATACAAATATATACAAAGGATCAGAATCCCTGGAAAAGTTAAATATATGGGTAAAAAGAAAAGATTTCTATTCTCACTTTTAATTTTCTTTGAAATGTAATTGGCTCTTTAAAGTAATCATAATGGCAGTATATTAGGGGATTTTTAATGTGTATATAGAAGAAAAATATATGAGAGTAATTGCCCAAAGATTAAACAGAGTATACAGTTACAACATTCTTATATTGTATGTGGGGTAGTATGGTATTATTTGTAGATAGACTGCAACAAGGTAAAGATGTATACTCTAAACTCCAGTGCAACCACTTTTAGAAAAAGAGAGAGACAGTTAACAAGCCAGTATTGGAGAAATAAGTGGAATATTTAAAAATGCTCAGTCTCAAAAGAGTCTGGAAAATAAGAACAAAGAATAGTGAAGACAAATAGAAAACAGATGGCAGAATGATAAACTCAATCATGTTGATAAATATATTAAATGTAAATGGCTTAAATATCCAACTAAAAGACTACTAGAATGGATTCTATTCTATTCTATTCTATTCTATTCTATTCTATTCTATTCTATTCTATTCTATTCTATTCTATTCTATTCTATTCTATTCTATTCTATTCTATTCTATTCTATTCTATTCTATTCTATTCTATTCTATTCTATTCTATTCTATTCTATTCTATTCTATTCTATTCTATTCTATTCTATTCTATTCTATTCTATTCTATTCTATTCTATTCTATTCTATTCTATTCTATTCTATTCTATTTTCTATTCTATTCTATTTTCTATTCTATTCTATTCTATTCTATTCTATTCTATTCTATTCTATTCTATTCTATTCTATTCTATTCTATTCTATTCTATTCTATTCTATTCTATTTTCTATTCTATTCTATTCTATTTTCTATTCTATTCTATTCTATTCTATTCTATTCTATTCTATTCTATTCTATTCTATTCTATTCTATTCTATTCTATTCTATTCTATTCTATTCTATTCTATTCTATTCTATTTTCTATTCTATTCTATTCTATTCTATTCTATTCTATTCTATTCTATTCTATTCTATTCTATTTTCTATTCTATTTTCTATTCTATTCTATTCTATTCTATTCTATTCTATTCTATTCTATTCTATTCTATTCTATTCTATTCTATTCTATTCTATTTTCTATTCTCTATTCTATTCTATTCTATTCTATTCTATTCTATTCTATTCTATTCTATTCTATTCTATTCTATTCTATTCTATTCTATTCTATTCTATTCTATTCTATTCTATATTCTATTCTATTCTATTCTATTCTATTCTATTCTATTCTATTCTATTCTATTCTATTTTCTATTCTATTCTATTCTATTTTCTATTCTATTCTATTCTATTCTATTCTATTCTATTCTATTCTATTCTATTCTATTCTATTCTATTTTCTATTCTATTCTATTCTATTTTCTATTCTATTCTATTCTATTCTATTCTATTCTATTCTATTCTATTCTATTCTATTCTATTTTCTATTCTATTCTATTCTATTTTCTATTCTATTCTATTCTATTTTCTATTCTATTCTATTCTATTCTATTCTATTCTATTCTATTCTATTCTATTCTATTCTATTCTATTCTATTCTATTTTCTATTCTATTTTCTATTCTATTCTATTCTATTCTATTCTATTCTATTCTATTCTATTCTATTCTATTCTATTCTATTCTATTCTATTTTCTATTCTATTTTCTATTCTATTTTCTATTCTATTTTCTATTCTATTTTCTATTCTATTCTATTCTATTCTATTCTATTCTATTCTATTCTATTCTATTCTATTTTCTATTCTATTTTCTATTCTATTCTATTCTATTCTATTCTATTCTATTCTATTCTATTCTATTCTATTCTATTCTATTCTATTTTCTATTCTATTTTCTATTCTATTTTCTATTCTATTTTCTATTCTATTCTATTCTATTCTATTCTATTCTATTCTATTCTATTCTATTCTATTCTATTCTATTCTATTTTCTATTCTATTTTCTATTCTATTTTCTATTCTATTTTCTATTCTATTTTCTATTCTATTTTCTATTCTATTTTCTATTCTATTTTCTATTCTATTTTCTATTCTATTTTCTATTCTATTTTCTATTCTATTTTCTATTCTATTCTATTCTATTCTATTCTATTCTATTCTATTCTATTCTATTCTATTCTATTTCTATTCTATTTCTATTCTATTCTATTCTATTCTATTCTATTCTATTCTATTCTATTCTATTCTATTCTATTCTATTCTATTCTATTCTATTCTATTCTATTCTATTCTATTCTATTCTATTCTATTCTATTCTATTCTATTCTATTCTATTCTATTCTATTCTATTCTATTCTATTCTATTCTATTCTATTCTATTCTATTCTATTCTATTCTATTCTATTCTATTCTATTCTATTCTATTCTATTCTATTCTATTCTATTCTATTCTATTCTATTCTATTCTATTCTATTCTATTCTATTCTATTCTATTCTATTCTATTCTATTCTATTCTATTCTATTCTATTCTATTCTATTCTATTCTATTCTATTCTATTCTATTCTATTCTATTCTATTCTATTCTATTCTATTCTATTCTATTCTATTCTATTCTATTCTATTCTATTCTATTCTATTCTATTCTATTCTATTCTATTCTATTCTATTCTATTCTATTCTATTCTATTCTATTCTATTCTATTCTATTCTATTCTATTCTATTCTATTCTATTCTATTCTATTCTATTCTATTCTATTCTATTCTATTCTATTCTATTCTATTCTATTCTATTCTATTCTATTCTATTCTATTCTATTCTATTCTATTCTATTCTATTCTATTCTATTCTATTCTATTCTATTCTATTCTATTCTATTCTATTCTATTCTATTCTATTCTATTCTATTCTATTCTATTCTATTCTATTCTATTCTATTCTATTCTATTCTATTCTATTCTATTCTATTCTATTCTATTCTATTCTATTCTATTCTATTCTATTCTATTCTATTCTATTCTATTCTATTCTATTCTATTCTATTCTATTCTATTCTATTCTATTCTATTCTATTCTATTCTATTCTATTCTATTCTATTCTATTCTATTCTATTCTATTCTATTCTATTCTATTCTATTCTATTCTATTCTATTCTATTCTATTCTATTCTATTCTATTCTATTCTATTCTATTCTATTCTATTCTATTCTATTCTATTCTATTCTATATTCTATTCTATTCTATTCTATATTCTATTCTATTCTATTCTATATTCTATTCTATTCTATATTCTATTCTATTCTATATTCTATTCTATTCTATATTCTATTCTATTCTATATTCTATTCTATTCTATATTCTATTCTATTCTATATTCTATTCTATTCTATATTCTATTCTATTCTATATTCTATTCTATTCTATATTCTATTCTATTCTATATTCTATTCTATTCTATATTCTATTCTATTCTATATTCTATTCTATTCTATATTCTATTCTATTCTATATTCTATTCTATTCTATATTCTATTCTATTCTCTATTCTATTCTATTCTCTATTCTATTCTATTCTCTATTCTATTCTATTCTCTATTCTATTCTATTCTCTATTCTATTCTATTCTCTATTCTATTCTATTCTCTATTCTATTCTATTCTCTATTCTATTCTATTCTCTATTCTATTCTATTCTCTATTCTATTCTATTCTCTATTCTATTCTATTCTCTATTCTATTCTATTCTCTATTCTATTCTATTCTCTATTCTATTCTATTCTCTATTCTATTCTATTCTCTATTCTATTCTATTCTCTATTCTATTCTATTCTCTATTCTATTCTATTCTCTATTCTATTCTATTCTCTATTCTATTCTATTCTCTATTCTATTCTATTCTCTATTCTATTCTATTCTCTATTCTATTCTATTCTCTATTCTATTCTATTCTCTATTCTATTCTATTCTCTATTCTATTCTATTCTCTATTCTATTCTATTCTATTCTATTCTATTCTATTCTATTCTATTCTATTCTATTCTATTCTATTCTATTCTATTCTATTCTATTCTATTCTATTCTATTCTATTCTATTCTATTCTATTCTATTCTATTCTATTCTATTCTATTCTATTCTATTCTATTCTATTCTATTCTATTCTATTCTATTCTATTCTATTCTATTCTATTCTATTCTATTCTATTCTATTCTATTCTATTCTATTCTATTCTATTCTATTCTATTCTATTCTATTCTATTCTATTCTATTCTATTCTATTCTATTCTATTCTATTCTATTCTATTCTATTCTATTCTATTCTATTCTATTCTATTCTATTCTATTCTATTCTATTCTATTCTATTCTATTCTATTCTATTCTATTCTATTCTATTCTATTCTATTCTATTCTATTCTATTCTATTCTATTCTATTCTATTCTATTCTATTCTATTCTATTCTATTCTATTCTATTCTATTCTATTCTATTCTATTCTATTCTATTCTATTCTATTCTATTCTATTCTATTCTATTCTATTCTATTCTATTCTATTCTATTCTATTCTATTCTATTCTATTCTATTCTATTCTATTCTATTCTATTCTATTCTATTCTATTCTATTCTATTCTATTCTATTCTATTCTATTCTATTCTATTCTATTCTATTCTATTCTATTCTATTCTATTCTATTCTATTCTATTCTATTCTATTCTATTCTATTCTATTCTATTCTATTCTATTCTATTCTATTCTATTCTATTCTATTCTATTCTATTCTATTCTATTCTATTCTATTCTATTCTATTCTATTCTATTCTATTCTATTCTTCTATTCTTTTTGAGATGAAGTCTCTCTATGTTGCCCAGGCTGGAGTGCAGTGGCGCGATCTCAGCTCACTGCAACCTCTGCCTACCAGGTTCAAGCAATTCTCCTGCCTCAGCCTCCCAAGTAGCTGAGATTACAGGTTCACCACCACACACCCAGCTAATTTTTGTATTTTTAGTAGAGACGGTGTTTCGGGAAGTCAGGGACCCTGAACGGAGGGACTGGCTGAAGCCATGGCAGAAGAACATAAATTGTTAAGATTTCATGGACATTTATTAGTTCCCCAAATTAATACTTTTATAATGTCTTACACCTGTCTTTACTGCAGTCTCTGAACATAAATTGTGAAGATTTCATGGACATTTATCACTTCCCTAATCAACACTCTTATAATTTCCTATGCCTGTCTTGTCTTTAATATCTTAATCTCGTCATCTTCATAAGCTGAGGATGTATGTCACCTCAGGATCCCGTGATGATCACGTTATCTGCACAAATTGTTTGTAAAGCATGTGTGTTTGAACAATATGAAATTTGGGCACCTTGAAAAAGAACTGGGTAACAGCGATTTTCA

>KM610328 (Subject B)

ATAACCAGCTTTTGGTTTTGGCTAATCTTCTCTGTAGTGTTGTTGTTCTAATTCACTGACTTTCTTGCCTGTCAGTTTATAAGAACAAAGATAAAAATGATAACAGGCTTTCCTTCAGAAATGATGCAAGACAGGAATCAATGGAATGACATCTTAAAAATACTAAAGGAAAACTGTTAATCTAGAATTATGTATCCAGCCAAAATATCCTTCAAAAATGAAGGTCAAAAAAAAGAAGGTAAAATGTCCTGTAAGAAATGTTAAATGAAGTTCAACAAGGTGAAAGTAAATGATAGCACATGGAAACCTGACCTATACAAATATATACAAAGGATCAGAATCCCTGGAAAAGTTAAAATATATGGGTAAAAAGAAAAGATTTCTATTCTCACTTTTAATTTTCTTTGAAATGTAATTGGCTCTTTAAAGTAATCATAATGGCAGTATATTAGGGGATTTTTAATGTGTATATAGAAGAAAAATATATGAGAGTAATTGCCCAAAGATTAAACAGAGTATACAGTTACAACATTCTTATATTGTATGTGGGTTAGTATGGTATTATTTGTAGATAGACTGCAACAAGGTAAAGATGTATACTCTAAACTCCAGTGCAACCACTTTTAGAAAAAGAGAGAGACAGTTAACAAGCCAGTATTGGAGAAATAAGTGGAATATTTTAAAAATGCTCAGTCTCAAAAGAGTCTGGAAAATAAGAACAAAGAATAGTGAAGACAAATAGAAAACAGATGGCAGAATGATAAACTCAATCATGTTGATAAATATATTAAATGTAAATGGCTTAAATATCCAACTAAAAGACTACTAGAATGGATTCTATTCTATTCTATTCTATTCTATTCTATTCTATTCTATTCTATTCTATTCTATTCTATTCTATTCTATTCTATTCTATTCTATTCTATTTCTATTTCTATTCTATTCTATTCTATTCTATTCTATTCTATTCTATTCTATTCTATTCTATTCTATTCTATTCTATTCTATTCTATTCTATTCTATTCTATATTCTATTCTATTCTATTCTATTCTATTCTATTCTATTCTATTTTCTATTCTATTCTAATTTCTATTCTATTCTATTCTATTCTATTCTATTCTATTCTATTCTATTCTATTCTATTCTATTCTATTCTATTCTATTCTATTCTATTCTATTCTATTCTATTCTATTCTATTCTATTCTATTCTATTCTATTCTATTCTATTCTATTCTATTCTATTCTATTCTATTCTATTCTATTCTATTCTATTCTATTCTATTCTATTCTATTCTATTCTATTCTATTCTATTCTATTCTATTCTATTCTATTCTATTCTATTCTATTCTATTCTATTCTATTCTATTCTATTCTATTCTATTCTATTCTATTCTATTCTATTCCTATTCTATTCTATTCTATTCTATTCTATTCTATTCTATTCTATTCTATTCTATTCTATTCTATTCTATTCTATTCTATTCTATTCTATTCTATTCTATTCTATTCTATTCTATTCTATTCTATTCTATTCTATTCTATTCTATTCTATTCTATTCTATTCTATTCTATTCTATTCTATTCTATTCTATTCTATTCTATTCTATTCTATTCTATTCTATTCTATTCTATTCTATTCTATTCTATTCTATTCTATTCTATTCTATTCTATTCTATTCTATTCTATTCTATTCTATTCTATTCTATTCTATTCTATTCTATTCTATTCTATTCTTACATTCTATTCTATTCTATTCTATTCTATTCTATTCTATTCTATTCTATTCTATTCTATTCTATTCTATTCTATTCTATTCTATTCTATTCTATTCTATTCTATTCTATTCTATTCTATTCTATTCTATTCTATTCTATTCTATTCTATTCTATTCTATTTCTATTCTATTCTAATTCTATTCTATTCTATTCTATTCTATTCTATTCTATTCTATTCTATTCTATTCTATTCTATTCTATTCTATTCTATTCTATTCTATTCTATTCTATTCTATTCTATTCTATTCTATTCTATTCTATTCTATTCTATTCTATTCTATTCTATTCTATTCTATTCTATTCTATTCTATTCTATTCTATTCTATTCTATTCTATTCTATTCTATTCTATTCTATTCTATTCTATTACTTCTATTCTATTCAATTCTATTCTATTCTATTCTATTCTATTCTATTCTATTCTATTCTATTTCTATTCTATTCTATTCTATTCTATTCTATTCTATTCTATTCTATTCTATTCTATTTCTATTCTATTCTATTCTATTCTATTCTATTCTATTCTATTCTTATTCTATTCTACTTTCTATTCTATTCTTCTTTCTATTCTATTCTATTCTATTCTATTCTATTCTATTCTATTCTATTCTATTCTATTCTATTCTATTCTATTCTATTCTATTCTATTCTATTCTATTCTATTCTATTCTATTTATTCTATTCTATTCTATTCTATTCTATTCTATTCTATTCTATTCTATTCTATTCTATTCTATTCTATTCTATTCTATTCTATTCTATTCTATTCTATTCTATTCTATTCTATTCTATTCTATTCTATTCTATTCTATTCTATTCTATTCTATTCTATTCTATTCTATTCTATTCTATTCTATTCTATTCTATTCTATTCTATTCTATTCTATTCTATTCTATTCTATTCTATTCTATTCTATTCTATTCTATTCTATTCTATTCTATTCTATTCTATTCTATTCTATTCTATTCTATTCTATTTCTATTCTATTCTATTCTATTCTATTCTATTCTATTCTATTCTATTCTATTCTATTCTATCTATTCTATTCTATTCTATTCTATTCTATTCTATTCTATTCTATTCTATTCTATTCTATTCTATTCTATTCTATTCTATTCTATTCTATTCTATTCTATTCTATTCTATTCTATTCTATTCTATTCTATTCTATTCTATTCTATTCTATTCTATTCTATTCTATTCTATTCTATTCTATTCTATTTTCTATTCTATTCTATTCTATTCTATTCTATTCTATTCTATTCTATTCCTATTCTATTCTATTCTATTCTATTCTATTCTATTCTATTCTATTCTATTCTATTCTATTCTATTCTATTCTATTCTATTTCTATTCTATTCTATTCTATTCTATTCTATTCTATTCTATTCTATTCTATTCTATTCTATTCTATTCTATTCTATTCTATTCTATTCTATTCTATTCTATTCTATTCTATTCTATTCTATTCTATTCTATTCTATTCTATTCTATTCTATTCTATTCTATTCTATTCTATTCTATTCTATTCTATTTCTATTCTATTCTATTCTATTCTATTCTATTCTATTCTATTCTATTCTATTCTATTCTATTCTATTCTATTCTATTCTATTCTATTCTATTCTATTCTATTCTATTTCTATTCTATTCTATTCTATTCTATTCTATTCTATTCTATTCTATTCTATTCTATTCTATTCTATTCTATTCTATTCTATTCTATTCTATTCTATTCTATTCTATTCTATTCTATTCTATTCTATTCTATTCTATATTCTATTCTATTCTATTCTATTCTATTCTATTCTATTCTATTCTATTCTATTCTATTCTATTCTATTCTATTCTATTCTATTTCTATTCTATTCTATTCTATTCTATTCTATTCTATTCTATTCTATTCCATTTCTATTCCATTCCTATTCCATTCTATCCATTCATTCCATTCCATTCCATTCCATTCCATTCCATTCCATTCCATTCCATTCCATTCCATTCCATTCCATTCCATTCCATTCCATTCCATTCCATTCCATTCCATTCCATTCCATTCCATTCCATTCCATTCCATTCCATTCCATTCCATTTCCATTCCATTCCATTCCATTCCCATTCCATTCCATTCCATTCCATTCCATTCCATTCCATTCCATTCCATTCCATTCCATTCCATTCCATTCCATTCCATTCCATTCCATTCCATTCCATTCCATTCCATTCCATTCCATTCCATTCCATTCCATTTCCATTCCATTCCATTCCATTCCATTCCATTCCATTCCTTCCCATTCCATTCCATTCCATTCCATTCCATTCCATTCCATTCCATTCCATTCCATTCCATTCCATTCCATTCCATTCCATTCCATTCCATTCCATTCCATTCCATTCCATTCCATTCCATTCCATTCCATTCCATTCCATTCCATTCCATTCCATTCCATTCCATTCCATTCCATTCCATTCCATTCCATTCCATTCCATTCCATTCCATTCCATTCCATTCCATTCCATTCCATTCCATTCCATTCCATTCCATTCCATTCCATTCCATTCCATTCCATTCCATTTTCCATTCCATTCCATTCCATTCCATTCCATTCCATTCCATTCCATTCCATTCCATTCCATTCCATTCCATTCCATTCCATTCCATTCCATTCCATTCCATTCCCATCCATTCCATTTCCATTCCATTCCATTCCATTCCATTCCATTCCATTCCATTCCATTCCATTCCATTCCATTCCATTCCATTCCATTCCATTCCATTCCATTCCATTCCATTCCATTCCATTCCATTCCATTCCATTCCATTCCATTCCATTCCATTCCATTCCCATTCCATTCCATTCCATTCCATTCCATTCCATTCCATTCCCATTCCATTCCATTCCATTCCATTCCATTCCATTCCATTCCATTCCATTCCCATTCCATTCCATTCCATTCCATTCCATTCCATTCCATTCCATTCCATTCCATTCCATTCCATTCCATTCCATTCCATTCCATTCCATTCCATTCCATTCCATTCCATTCCATTCCATTCCATTCCATTCCCATTCCATTCCATTCATTCCATTCCATTCCATTCCATTCCATTCCATTCCATTCCATTCCATTTCCATTCCATTCCATTCCATTCCATTCCATTCCATTCCATTCCATTCCATTTCCATTCCATTCCATTCCATTCCATTCCATTCCATTCCATTCCATTCCATTCCATTCCATTCCATTCCATTCATTCCATTCCATTCCATTCCATTCCATTCCATTCCATTCCATTCCATTCCATTCCATTCCATTCCATTCTATTCTATTCTATTCTATTCTATTCTATTCTATTCTATTCTTTTTTTTGAGATGAAGTCTCTCTATGTTGCCCAAGGCTGGAGTGCAGTGGCGCGATCTCAGCTCACTGCAACCTCTGCCTACCAGGTTCAAGCAATTCTCCTGCCTCAGCCTCCCAAGTAGCTGAGATTACAGGTTCACCACCACACCCAGCTAATTTTTGTATTTTTAGTAGAGACGGTGTTTTCGGGAAGTCAGGGACCCTGAACGGAGGGACTGGCTGAAGCCATGGCAGAAGAACATAAATTGTTAAGATTTCATGGACATTTATTAGTTCCCCAAATTAATACTTTTATAATGTCTTACACCTGTCTTTACTGCAGTCTCTGAACATAAACTGTGAAGATTTCATGGACATTTATCACTTCCCTAATCAACACTCTTATAATTTCCTATGCCTGTCTTGTCTTTAATATCTTAATCTCGTCATCTTCATAAGCTGAGGATGTATGTCACCTCAGGATCCCGTGATGATCACGTTATCTGCACAAATTGTTTGTAAAGCATGTGTGTTTGAACAATATGAAATTTGGGCACCTTGAAAAAGAACTGGGTAACAGCGATTTTCAAGGAACAAGGGAGATAACCATAAGGCCTGACTGCCTGCAGGGCCGGGCAGAACAGAGTCATATTTCTCTTCTTTCAGAAAGTGACTAGGAGAAATATCGCTGAATTCTTTTCTCAGCAAGGAATAACCCTGG

>KM610329 (Subject C)

TCTAGAATTATGTATCCAGCCAAAATATCCTTCAAAAATGAAGGTCAAAAAAAAGAAGGTAAAATGTCCTGTAAGAAATGTTAAATGAAGTTCAACAAGGTGAAAGTAAATGATAGCACATGGAAACCTGACCTATACAAATATATACAAAGGATCAGAATCCCTGGAAAAGTTAAATATATGGGTAAAAAGAAAAGATTTCTATTCTCACTTTTAATTTTCTTTGAAATGTAATTGGCTCTTTAAAGTAATCATAATGGCAGTATATTAGGGATTTTTAATGTGTATATAGAAGAAAAATATATGAGAGTAATTGCCCAAAGATTAAACAGAGTATACAGTTACAACATTCTTATATTGTATGTGGGGTAGTATGGTATTATTTGTAGATAGACTGCAACAAGGTAAAGATGTATACTCTAAACTCCAGTGCAACCACTTTTAGAAAAAGAGAGAGACAGTTAACAAGCCAGTATTGGAGAAATAAGTGGAATATTTAAAAATGCTCAGTCTCAAAAGAGTCTGGAAAATAAGAACAAAGAATAGTGAAGACAAATAGAAAACAGATGGCAGAATGATAAACTCAATCATGTTGATAAATATATTAAATGTAAATGGCTTAAATATCCAACTAAAAGACTACTAGAATGGATTCTATTCTATTCTATTCTATTCTATCTATTCTATTCTATTCTATTCTATTCTATTCTATTCTATTCTATTCTATTCTATTCTATTCTATTCTATTCTATTCTATTCTATTCTATCTATTCTATTCTATTCTATTCTATTCTATTCTATTCTATTCTATCTATTCTATTCTATTCTATTCTATTCTATTCTATTCTATTCTATTCTATTCTATTTTCTATTCTATATTCTATTCTATATTCTATTCTATTTTCTATTCTATTCTATTCTATTCTATTCTATTCTATTCTATTCTATTCTATTCTATTCTATATTCTATTCTATTCTATTCTATTCTATTCTATATTCTATTCTATTCTATTCTATTCTATTCTATTCTATTCTATTCTATTCTATTCTATATTCTATTCTATTCTATATTCTATTCTATTCTATTCTATTCTATTCTATTCTATTCTATTCTATTCTATTCTATTCTATATTCTATTCTATATTCTATTCTATTCTATTCTATTCTATTCTATTCTATTCTATTCTATTCTATTCTATTCTATATTCTATTCTATATTCTATTCTATATTCTATTCTATATTCTATTCTATTCTATTCTATATTCTATTCTATTCTATTCTATTCTATTCTATTCTATTCTATTCTATTCTATTCTATATTCTATTCTATATTCTATTCTATATTCTATTCTATATTCTATTCTATATTCTATTCTATTCTATTCTATTCTATTCTATTCTATTCTATTCTATATTCTATTCTATTCTATTCTATTCTATTCTATTCTATTCTATTCTATTCTATTCTATTCTATTCTATTCTATATTCTATTCTATTCTATTCTATATTCTATTCTATTCTATTCTATTCTATTCTATTCTATTCTATTCTATTCTATTCTATTCTATTCTATTCTATTCTATTCTATTCTATTCTATTCTATCCTATCCTATCCTATCCTATCCTATCCTATCCTATCCTATCCTATCCTATCCTATCCTATCCTATCCTATCCTATCCTATATCCTATCCTATCCTATCCTATCCTATCCTATCCTATCCTATCCTATCCTATCCTATCTATCCTATCCTATCCTATCCTATCCCTATCCTATCCTATCCTATCCTATCCTATCATCCATCCCATCCCATCCCATCCCATCCATCCCATCCCATCCCATCCCATCCCATCCATCCCATCCCATCCCATCCCATCCCATCCCATCCCATCCCATCCCATCCCATCCCATCCCATCCATTCCATCCCATCCCATCCCATCCCATCCCATCCATCCCATCCCATCCCATCCCATCCATCCCATCCCATCCCATCCCATCCCATCCCATCCCATCCCATCCCATCCCATCCCATCCCATCCCATCCCATCCCATCCCATCCCATCCCATCCATCCCATCCATCCCATCCCATCCCATCCCATCCCATCCCATCCCATCCCATCCCATCCCATCCCATCCCATCCCATCCCATCCCATCCATCCCATCCCATCCCATCCCATCCCATCCCCATCCCATCCCATCCATCCCATCCCATCCCATCCCATCCCATCCCATCCCATCCCATCCCATCCCATCCCATCCCATCCCATCCATCCCATCCCATCCCATCCCATCCCATCCCATCCCATCCATCCCATCCCATCCCATCCATCCCATCCCATCCCATCCCATCCCATCCATCCCATCCCATCCCATCCCATCCATCCCATCCCATCCCATCCCATCCCATCCCATCCCATCCCATCCCATCCCATCCCATCCCATCCCATCCCATCCATCCCATCCCATCATCCATCCCATCCCATCCCATCCCATCCCATCCCATCCCATCCCATCCCATCCCATCCCATCCCATCCCATCCCATCCATCCCATCCCATCCCATCCCATCCCATCCCATCCCATCCCATCCCATCCCATCCCATCCCATCCCATCCCATCCCATCCCATCCCATCCCATCCCATCCCATCCCATCCCATCCCATCCCATCCCATCCCATCCCATCCCATCCCATCCCATCCCATCCCATCCCATCCCATCCCATCCCATCCCATCCCATCCCATCCATCCCATCCCATCCATCCCATCCCATCCCATCCCATCCCATCCCATCCCATCCATCCCATCCCATCCCATCCCATCCCATCCCATCCCATCCCATCCCATCCCATCCCATCCCATCCATCCCATCCCATCCCATCCCATCCCATCCCATCCCATCCCATCCCATCCCATCCCATCCCATCCCATCCCATCCCATCCCATCCCATCCCATCCCATCCCATCCCATCCCATCCCATCCCATCCCATCCCATCCCATCCCATCCCATCCCATCCCATCCCATCCCATCCCATCCCATCCCATCCCATCCCATCCCATCCCATCCATCCCATCCCATCCCATCCCATCCCATCCCATCCCATCCCATCCCATCCCATCCCATCCCATCCCATCCCATCCATCCCATCCCATCCCATCCCATCCCATCCCATCCCATCCCCATCCCATCCCATCCCATCCCATCCTATCCTATCCTATTCTTTTTGAGATGAAGTCTCTCTATGTTGCCCAGGCTGGAGTGCAGTGGCGCGATCTCAGCTCACTGCAACCTCTGCCTACCAGGTTCAAGCAATTCTCCTGCCTCAGTCTCCCAAGTAGCTGAGATTACAGGTTCACCACCACACCCAGCTAATTTTTGTATTTTTAGTAGAGACGGTGTTTCGGGAAGTCAGGGACCCTGAACGGAGGGACTGGCTGAAGCCATGGCAGAAGAACATAAATTGTTAAGATTTCATGGACATTTATTAGTTCCCCAAATTAATACTTTTATAATGTCTTACACCTGTCTTTACTGCAGTCTCTGAACATAAATTGTGAAGATTTCATGGACATTTATCACTTCCCTAATCAACACTCTTATAATTTCCTATGCCTGTCTTGTCTTTAATATCTTAATCTCGTCATCTTCATAAGCTGAGGATGTATGTCACCTCAG

>Subject C, clone 2 (KT223133)

TCTAGAATTATGTATCCAGCCAAAATATCCTTCAAAAATGAAGGTCAAAAAAAAGAAGGTAAAATGTCCTGTAAGAAATGTTAAATGAAGTTCAACAAGGTGAAAGTAAATGATAGCACATGGAAACCTGACCTATACAAATATATACAAAGGATCAGAATCCCTGGAAAAGTTAAATATATGGGTAAAAAGAAAAGATTTCTATTCTCACTTTTAATTTTCTTTGAAATGTAATTGGCTCTTTAAAGTAATCATAATGGCAGTATATTAGGGGATTTTTAATGTGTATATAGAAGAAAAATATATGAGAGTAATTGCCCAAAGATTAAACAGAGTATACAGTTACAACATTCTTATATTGTATGTGGGGTAGTATGGTATTATTTGTAGATAGACTGCAACAAGGTAAAGATGTATACTCTAAACTCCAGTGCAACCACTTTTAGAAAAAGAGAGAGACAGTTAACAAGCCAGTATTGGAGAAATAAGTGGAATATTTAAAAATGCTCAGTCTCAAAAGAGTCTGGAAAATAAGAACAAAGAATAGTGAAGACAAATAGAAAACAGATGGCAGAATGATAAACTCAATCATGTTGATAAATATATTAAATGTAAATGGCTTAAATATCCAACTAAAAGACTACTAGAATGGATTCTATTCTATTCTATTCTATTCTATTCTATTCTATTCTATTCTATTCTATTCTATTCTATTCTATTCTATTCTATTCTATTCTATTCTATTCTATTCTATTCTATTCTATTCTATTCTATTCTATTCTATTCTATTCTATTCTATTCTATTCTATTCTATTCTATTCTATTCTATTCTATTCTATTCTATTCTATTCTATTCTATTCTATTTTCTATTCTATATTCTATTCTATATTCTATTCTATTTTCTATTCTATTCTATTCTATTCTATTCTATTCTATTCTATTCTATTCTATTCTATTCTATATTCTATTCTATTCTATTCTATTCTATTCTATTCTATTCTATTCTATTCTATTCTATTCTATTCTATTCTATTCTATTCTATTCTATTCTATTCTATTCTATATTCTATTCTATATTCTATTCTATTCTATTCTATTCTATTCTATTCTATTCTATTCTATTCTATTCTATTCTATATTCTATTCTATATTCTATTCTATATTCTATTCTATATTCTATTCTATTCTATTCTATATTCTATTCTATTCTATTCTATTCTATTCTATTCTATTCTATTCTATTCTATTCTATATTCTATTCTATATTCTATTCTATATTCTATTCTATATTCTATTCTATATTCTATTCTATTCTATTCTATTCTATTCTATTCTATTCTATTCTATATTCTATTCTATTCTATTCTATTCTATTCTATTCTATTCTATTCTATTCTATTCTATTCTATTCTATTCTATATTCTATTCTATTCTATTCTATATTCTATTCTATTCTATTCTATTCTATTCTATTCTATTCTATTCTATTCTATTCTATTCTATTCTATTCTATTCTATTCTATTCTATTCTATTCTATCCTATCCTATCCTATCCTATCCTATCCTATCCTATCCTATCCTATCCTATCCTATCCTATCCTATCCTATCCTATCCTATCCTATCCTATCCTATCCTATCCTATCCTATCCTATCCTATCCTATCCTATCCTATCCTATCCTATCCTATCCTATCCTATCCTATCCTATCCTATCCTATCCTATCCCATCCCATCCCATCCCATCCCATCCCATCCCATCCCATCCCATCCCATCCCATCCCATCCCATCCCATCCCATCCCATCCCATCCCATCCCATCCCATCCCATCCCATCCCATCCCATCCCATCCCATTCCATCCCATCCCATCCCATCCCATCCCATCCCATCCCATCCCATCCCATCCCATCCCATCCCATCCCATCCCATCCCATCCCATCCCATCCCATCCCATCCCATCCCATCCCATCCCATCCCATCCCATCCCATCCCATCCCATCCCATCCCATCCCATCCCATCCCATCCCATCCCATCCCATCCCATCCCATCCCATCCCATCCCATCCCATCCCATCCCATCCCATCCCATCCCATCCCATCCCATCCCATCCCATCCCATCCCATCCCATCCCATCCCATCCCATCCCATCCCATCCCATCCCATCCCATCCCATCCCATCCCATCCCATCCCATCCCATCCCATCCCATCCCATCCCATCCCATCCCATCCCATCCCATCCCATCCCATCCCATCCCATCCCATCCCATCCCATCCCATCCCATCCCATCCCATCCCATCCCATCCCATCCCATCCCATCCCATCCCATCCCATCCCATCCCATCCCATCCCATCCCATCCCATCCCATCCCATCCCATCCCATCCCATCCCATCCCATCCCATCCCATCCCATCCCATCCCATCCCATCCCATCCCATCCCATCCCATCCCATCCCATCCCATCCCATCCCATCCCATCCCATCCCATCCCATCCCATCCCATCCCATCCCACCCCATCCCATCCCATCCCATCCCATCCCATCCCATCCCATCCCATCCCATCCCATCCCATCCCATCCCATCCCATCCCATCCCATCCCATCCCATCCCATCCCATCCCATCCCATCCCATCCCATCCCATCCCATCCCATCCCATCCCATCCCATCCCATCCCATCCCATCCCATCCCATCCCATCCCATCCCATCCCATCCCATCCCATCCCATCCCATCCCATTCCATCCCATCCCATCCCATCCCATCCCATCCCATCCCATCCCATCCCATCCCATCCCATCCCATCCCATCCCATCCCATCCCATCCCATCCCATCCCATCCCATCCCATCCCATCCCATCCCATCCCATCCCATCCCATCCCATCCCATCCCATCCCATCCCATCCCATCCCATCCCATCCCATCCCATCCCATCCCATCCCATCCCATCCCATCCCATCCCATCCCATCCCATCCCATCCCATCCCATCCCATCCCATCCCATCCCATCCCATCCCATCCCATCCCATCCCATCCCATCCCATCCCATCCCATCCCATCCCATCCCATCCCATCCCATCCCATCCCATCCCATCCCATCCCATCCCATCCCATCCCATCCCATCCCATCCCATCCCATCCCATCCCATCCCATCCTATCCTATCCTATTCTTTTTGAGATGAAGTCTCTCTATGTTGCCCAGGCTGGAGTGCAGTGGCGCGATCTCAGCTCACTGCAACCTCTGCCTACCAGGTTCAAGCAATTCTCCTGCCTCAGTCTCCCAAGTAGCTGAGATTACAGGTTCACCACCACACCCAGCTAATTTTTGTATTTTTAGTAGAGACGGTGTTTCGGGAAGTCAGGGACCCTGAACGGAGGGACTGGCTGAAGCCATGGCAGAAGAACATAAATTGTTAAGATTTCATGGACATTTATTAGTTCCCCAAATTAATACTTTTATAATGTCTTACACCTGTCTTTACTGCAGTCTCTGAACATAAATTGTGAAGATTTCATGGACATTTATCACTTCCCTAATCAACACTCTTATAATTTCCTATGCCTGTCTTGTCTTTAATATCTTAATCTCGTCATCTTCATAAGCTGAGGATGTATGTCACCTCAG
